# Beyond Pathology: Bringing the Ecological-Enactive Model of Disability to Neuroethics and Mental Health Conditions

**DOI:** 10.1080/21507740.2024.2438005

**Published:** 2025-01-13

**Authors:** Andrew J. Barnhart

**Affiliations:** University of Bonn

The discourse surrounding mental health conditions (MHCs) can fluctuate between versions of the medical model, which views such conditions as pathologies requiring clinical intervention, and the neurodiversity model, which emphasizes acceptance and variation among individuals. A lot of ink has been spilled in critiquing these models of disability, even in relation to MHCs. Medical and neurodiversity models raise both conceptual and value conflicts between clinicians and disability advocates, making it difficult to establish common ground and dialogue (Barnhart and Dierickx 2021). Furthermore, both models arguably fail to recognize or fully capture the lived embodiment of individuals with disabilities (Toro, Kiverstein, and Rietveld 2020). As highlighted in Julia Knopes’s (2025) recent exploration of peer providers’ narratives, there is an expressed ambivalence toward traditional models, often borrowing language and concepts from medical or neurodiversity frameworks. Knopes (2025, 10) concludes that “This does not suggest that the models are flawed and futile, but rather, that people find different models applicable to different dimensions of their lived experiences.” While I do not contest the narratives of the peer providers themselves, I do suggest that the ambivalence, language, and conceptual borrowing from the more traditional models is further evidence that these models do fail to capture the lived experience of disability. Meanwhile, newer models are seeking to provide a solid the ethical foundation to disability narratives. One such model is the ecological-enactive model of disability, which reframes disability not as a static pathology or social difference but as a dynamic interplay between individuals and their environments, emphasizing the capacity for adaptation and interaction (Toro, Kiverstein, and Rietveld 2020; Heras-Escribano 2021; Schwab et al. 2022; Jurgens 2023; Nešić 2023). The model draws from both ecological psychology and enactive cognitive science (Toro, Kiverstein, and Rietveld 2020; Schwab et al. 2022; Jurgens 2023). This perspective can not only respect the fluid and context-dependent nature of MHCs, but also challenge the pathologization and reductionism inherent in traditional models. The model also has some empirical backing via interview studies with participants with Friedreich’s ataxia and cerebral palsy (Toro, Kiverstein, and Rietveld 2020; Schwab et al. 2022). This model can serve as a basis for creating and deepening more nuanced neuroethical discussions for individuals with MHCs. It could foster more inclusive and adaptive mental health practices that honor individual experiences, thereby enhancing both clinical and ethical strategies in mental health care.

At the heart of the ecological-enactive model is the concept of affordances—the possibilities for action that the environment offers—which individuals can utilize based on their skills and abilities (Toro, Kiverstein, and Rietveld 2020). Individuals directly perceive and respond to these affordances, engaging in a continuous process of adaptation that creates what is termed dynamic stability—a state of equilibrium with the environment achieved through ongoing interaction and adjustment.

Dynamic stability is a crucial part of the ecological-enactive model, where normal embodiment is characterized by an individual’s ability to “maintain a state of dynamic stability” despite the challenges posed by daily life (Toro, Kiverstein, and Rietveld 2020, 7). Furthermore, normal embodiment does not entail the absence of difficulty but rather reflects an individual’s capacity to navigate and adapt to these daily challenges by effectively leveraging available affordances (Schwab et al. 2022). It is this adaptability that is the hallmark of a healthy interaction with the environment, allowing individuals to respond flexibly to the demands of their surroundings.

Consider a person with bipolar disorder who has identified specific environmental and lifestyle factors that help them maintain dynamic stability and harness unique cognitive strengths associated with their condition. This individual might work in the creative industry where their ability to think divergently and generate innovative ideas is highly valued. The workplace provides a flexible schedule, allowing them to work during their most productive times, which aligns with their natural energy fluctuations. To maintain dynamic stability, the individual has established a routine that includes regular sleep patterns, a balanced diet, and mindfulness practices, which are affordances that help regulate mood swings. They also have a supportive network of colleagues and friends who understand their condition and provide encouragement and assistance when needed. In this scenario, the individual perceives and utilizes these affordances to navigate the challenges of bipolar disorder effectively. They can adapt to changes in their mood by leveraging the supportive elements of their environment, such as taking breaks when feeling overwhelmed or engaging in creative tasks during periods of high energy. This adaptability and proactive engagement with their environment exemplify normal embodiment, as they are not merely coping with their condition but actively shaping their interactions with the world to maintain equilibrium and enhance their well-being.

In contrast, pathological embodiment emerges when an individual’s interactions with their environment lead to persistent maladaptive responses leading to an inability to maintain dynamic stability, often due to a misalignment between environmental affordances and the individual’s capabilities. This misalignment creates a pervasive sense of “I-cannot,” a feeling of limitation and incapacity that can contribute to the development of further MHCs, such as anxiety or depression, which in turn may lead to avoidance behaviors and further withdrawal from beneficial environmental interactions (Toro, Kiverstein, and Rietveld 2020; Schwab et al. 2022). Moreover, pathological embodiment of other conditions or impairments could lead to further MHCs like anxiety or depression, which would lead to further avoidance behaviors (Jurgens 2023).

Consider again a person with bipolar disorder who experiences extreme mood fluctuations that significantly impact their ability to engage with their environment. During depressive episodes, the individual might find it challenging to perceive and utilize the affordances in their environment that could help stabilize their mood, such as social support or engaging in activities that typically bring joy or satisfaction. The environment, in this case, fails to provide opportunities that align with the individual’s current capabilities, leading to withdrawal and isolation. For instance, this person might have a supportive network of friends and family, but during depressive phases, they perceive social interactions as overwhelming or burdensome, leading them to avoid these interactions despite their potential benefits. This avoidance behavior can exacerbate feelings of loneliness and helplessness, reinforcing the sense of “I-cannot” engage with the world effectively. Similarly, during manic episodes, the individual might engage in risky behaviors without adequately considering the consequences, as the environment’s affordances for excitement and stimulation are perceived as overwhelmingly positive, overshadowing potential risks. This misalignment between the individual’s actions and the environment’s affordances can lead to negative outcomes, such as financial difficulties or damaged relationships, further contributing to a cycle of maladaptive responses.

However, under the ecological-enactive model, disability does not necessitate a pathological embodiment since not all impairments or conditions lead to a reduction of, or withdrawal from, the environment (Toro, Kiverstein, and Rietveld 2020; Jurgens 2023). Moreover, the ecological-enactive model supports the idea that some individuals may find value in their MHCs, as illustrated by Knopes (2025, 25), who reports participants who suggest “having a mental health condition can at times be less distressing (and carry some advantages) than partaking in the associated treatments.” For example, an individual with bipolar disorder may experience heightened creativity and periods of intense focus during manic episodes, which they might use to their advantage in artistic or professional pursuits, thereby finding a unique value in their condition that traditional models might overlook.

What does the ecological-enactive model ultimately mean for neuroethics and MHCs? Theoretically, it reframes mental health not as a series of pathologies but as adaptive interactions with one’s environment and challenges the reductionist tendencies of other frameworks. Furthermore, the ecological-enactive model invites a reevaluation of ethical standards in mental health care. It suggests that interventions should not only aim to alter individual states but also consider modifying environmental affordances to enhance well-being. To borrow from previous considerations of the ecological-enactive model for autism, it suggests that if MHCs are not something that can be (or should be) eradicated, then the aim should be to modify environmental affordances to accommodate them (Nešić 2023).

None of this is to say that peer-providers or individuals with MHCs should immediately adopt the rather technical language of the ecological-enactive model for their own narratives. They might still reach toward elements of more traditional models that are readily available and well known for themselves (perhaps preferring ambivalent practicality over enthusiastic complexity when narrative constructing). Indeed, as Knopes (2025) suggests, clinicians should be prepared for patients describing their MHCs as forms of neurodiversity worthy of value. But reaching for parts of old models to help build narratives does not mean that the models are inherently valuable in providing explanations on the origins, demarcations, or nature of disability or MHCs. Knopes is correct in that there is a need for a more expansive and inclusive view of MHCs, and hopefully the ecological-enactive model can help make this happen.
